# Low-Dose Prophylactic Oral Iron Supplementation (Ferrous Fumarate, Ferrous Bisglycinate, and Ferrous Sulphate) in Pregnancy Is Not Associated With Clinically Significant Gastrointestinal Complaints: Results From Two Randomized Studies

**DOI:** 10.1155/2024/1716798

**Published:** 2024-11-15

**Authors:** Nils Thorm Milman, Thomas Bergholt

**Affiliations:** ^1^Department of Obstetrics and Gynecology, Herlev and Gentofte University Hospital DK-2730, Herlev, Denmark; ^2^Department of Clinical Medicine, Faculty of Health and Medical Sciences, University of Copenhagen DK-2200, Copenhagen N, Denmark

**Keywords:** ferrous bisglycinate, ferrous fumarate, ferrous sulphate, iron, iron deficiency, pregnancy, prophylaxis, side effects, supplement

## Abstract

**Background:** Many pregnant women are reluctant to follow the recommendation concerning oral iron prophylaxis due to concerns about gastrointestinal (GI) side effects.

**Objective:** To assess the frequency of GI complaints during low-dose oral iron prophylaxis and compare three iron formulas in equipotent doses: ferrous fumarate versus ferrous bisglycinate versus ferrous sulphate, in healthy women with an uncomplicated single pregnancy.

**Methods:** Results from two randomized, double-blind studies are reported: the Gentofte study comprising 404 women allocated into four groups taking 20, 40, 60, and 80 mg of elemental iron as ferrous fumarate/day and the Naestved study comprising 78 women allocated into two groups: 25 mg of elemental iron as ferrous bisglycinate/day and 50 mg of elemental iron as ferrous sulphate/day between meals from 15 to 19 weeks of gestation to delivery. GI complaints (nausea, vomiting, epigastric pain/pyrosis, belching, meteorism, borborygmi, intestinal colic, flatulence, loose stools, constipation, and use of laxatives), as well as black stools, were recorded by interview at the time of inclusion and at regular intervals during gestation.

**Results:** At inclusion, the frequency of total combined GI complaints in all women (*n* = 482) was 21%. The Gentofte study showed that in the groups taking 20–60 mg iron/day as fumarate, there was no association between the iron dose and the frequency of GI side effects. An iron dose of 80 mg as fumarate was associated with significantly higher frequencies of constipation and the use of laxatives. Comparing the three equipotent doses of iron formulas, which can prevent iron deficiency, ferrous bisglycinate 25 mg iron had the most favourable GI side effect profile, while ferrous fumarate 40 mg iron and ferrous sulphate 50 mg iron had higher but similar GI side effect profiles. The frequency of black stools increased with the iron dose. Ferrous bisglycinate 25 mg iron had a lower frequency of black stools (8%) than ferrous fumarate 40 mg iron (22%) and ferrous sulphate 50 mg iron (31%).

**Conclusion:** Low-dose iron supplementation appears to have no clinically significant GI side effects, as none of the included women presented with GI complaints of such severity that it necessitated either reduction of iron dose, change to an alternative iron formula, or discontinuation of iron supplement. However, ferrous bisglycinate 25 mg iron/day is associated with significantly fewer GI complaints than ferrous fumarate 40 mg iron/day and ferrous sulphate 50 mg iron/day. Ferrous bisglycinate may be preferred for iron prophylaxis, especially in women experiencing GI side effects when taking other conventional iron formulas.

## 1. Introduction

During pregnancy, the demand for iron is markedly increased due to the expansion of the pregnant woman's hemoglobin mass and the concomitant need for iron in the growing fetus [1]. In many European and developed countries, oral iron supplementation is now routinely recommended to pregnant women because studies have shown that women not taking iron supplements suffer a high frequency of iron deficiency (ID) and many display ID anemia (IDA) [2]. Therefore, since 1992, the Danish Health Authority has recommended general oral iron prophylaxis to pregnant women from their first visit to the antenatal clinic [3]. A Danish randomized study found that a low dose (i.e., a dose being considerably lower than the dose used for treatment of IDA) of 40 mg of elemental iron as ferrous fumarate daily is sufficient to prevent ID and IDA in 90% and 95%, respectively, of all pregnancies [2]. Thus, in 2013, the Danish Health Authorities changed their recommendation on prophylactic oral iron supplementation from 50–70 mg of elemental ferrous iron/day to 40–50 mg/day [3] without recommending any specific iron formula.

However, many women are still reluctant to follow the recommendation, as they are worried about gastrointestinal (GI) discomfort or side effects of oral iron supplements. The percentage of women in Denmark complying with the iron prophylaxis recommendation in the first trimester varies from 77% [4] to 50% [5], probably due to differences in study designs and in the ethnic composition of the cohorts.

Uncomplicated pregnancy is accompanied by “physiological” GI discomfort, with considerable individual variation between the women [6–8]. The GI complaints are partly due to hormonal changes, for example, increased production of progesterone inhibiting GI motility resulting in constipation, increased levels of prostaglandins causing diarrhea [6, 7], and increased fetoplacental production of GDF15, which appears to be linked to the maternal risk of nausea and vomiting during pregnancy [8]. Furthermore, the pressure of the growing uterus on the abdominal organs, the ventricle, and the intestines provokes gastroesophageal reflux with symptoms such as epigastric pain, pyrosis, heartburn, cardialgia, and constipation [6, 7].

Due to these physiologically induced changes in GI function in uncomplicated pregnancy, it is difficult to evaluate the possible GI side effects of oral iron supplements during gestation.

This paper is based on two previously published investigations of prophylactic oral iron supplementation in healthy ethnic Danish pregnant women, termed the Gentofte study [2] and the Naestved study [9], respectively, of which the details have been described elsewhere. In both studies, the GI complaints were recorded, but they have not been reported and analyzed in detail. GI complaints in the Gentofte study [2] have previously been presented in an abbreviated version [10] with a statistical approach using the percentages of positive responders in each type of GI complaint. However, now, it is not generally recommended to cite percentages associated with outcomes in qualitative research. Consequently, in the present paper, we have used the actual number of positive responders in relation to the total number of women who answered the specific question, allowing us to perform more detailed comparative analyses between groups. Furthermore, in previous statistics, we included black stools as a side effect, but this is not the case in the present study.

The Gentofte study [2] was a randomized, double-blind, dose-response study with the intention to determine the lowest dose of elemental iron (as ferrous fumarate) being sufficient to prevent ID and IDA during pregnancy. The women were consecutively randomized into four groups taking 20, 40, 60, and 80 mg of elemental iron, respectively, from 17 to 18 weeks of gestation to delivery. For ethical reasons, a placebo group was not included in the study design.

The Naestved study [9] was a randomized, double-blind, parallel study comparing the effects on iron status of ferrous bisglycinate 25 mg of elemental iron and ferrous sulphate 50 mg of elemental iron, respectively, taken from 15 to 19 weeks of gestation to delivery. The present paper presents the analysis of GI complaints in the two series both separately and combined using a different angle of perspective and statistical analysis, which has not previously been published.

In both studies [2, 9], GI complaints were recorded on a questionnaire designed by the authors specifically for these studies, as there was no standardized questionnaire available when the studies were planned.

There exist few randomized, controlled studies of the potential GI side effects of oral iron supplementation in pregnancy [11]. Therefore, the aim of the present paper was twofold: first to assess the frequency of clinically significant GI side effects (i.e., complaints that necessitate reduction of iron dose, change to an alternative iron formula, or discontinuation of iron supplement) during iron prophylaxis in relation to increasing doses of ferrous fumarate and secondly to compare the potential GI side effects of three different iron formulas (ferrous fumarate, ferrous bisglycinate, and ferrous sulphate) in low doses (i.e., doses being considerably lower than the doses used for treatment of IDA), which are commonly used as prophylactic iron supplements in pregnant women in Denmark.

## 2. Materials and Methods

### 2.1. Women

All women in both studies fulfilled the following criteria: healthy ethnic Danish women > 18 years of age, with an uncomplicated single pregnancy. Previous pregnancies and deliveries should be uncomplicated.

The participants in the Gentofte study [2] were urban residents in Gentofte and adjacent municipalities in Greater Copenhagen, being a wealthy region of the city with middle- and high-income citizens. A total of 404 women with a mean age of 30 years were included consecutively at 17–18 weeks of gestation and randomized into the four iron groups. The women were told to take the tablets daily at bedtime or alternatively between meals. During the study, there were dropouts, as previously reported [2].

The participants in the Naestved study [9] were residents in the southwestern part of Region Zealand, consisting of a predominantly low- and middle-income rural population. A total of 80 women with a mean age of 28 years were included consecutively at 15–19 weeks of gestation and randomized in two groups taking either ferrous bisglycinate 25 mg of elemental iron/day (Aminojern) (“bisglycinate group”) or ferrous sulphate 50 mg of elemental iron mg/day (“sulphate group”) from inclusion until delivery. The women were told to take the tablets daily at bedtime or alternatively between meals.

At inclusion, the majority of women were taking various formulas of multivitamin–multimineral and iron supplements, containing from 9 to 100 mg of elemental ferrous iron, which were discontinued. The women were provided with daily iron as well as multivitamin–multimineral tablets without iron and containing folic acid and vitamin B_12_.

The frequencies of GI discomfort/complaints (nausea, vomiting, epigastric pain/pyrosis, belching, meteorism, borborygmi, intestinal colic, flatulence, loose stools, constipation, and the use of laxatives) and black stools were recorded by interview at each visit by the midwife using the same questionnaire in both studies. The participant's response to the questions should be yes or no and could not be graduated. The occurrence of black stools can be an aesthetic challenge to the woman and may have a negative influence on compliance with iron supplementation. However, it is a “physiological” consequence of oral iron supplementation, which depends both on the iron dose and the iron formula. Therefore, in contrast to our previous paper [10], the frequency of black stools is not registered as a GI complaint but is reported separately. Generally, the individual woman was followed by the same midwife during the entire pregnancy.

Overall, there was a high compliance with iron supplementation in both studies. In the Gentofte study, women taking less than 90% of the supplied tablets were excluded from the study (*n* = 30). In the Naestved study, the “compliance index” was calculated as the number of taken tablets/number of tablets that should have been taken if the compliance was 100%. The mean compliance index was 0.89 ± 0.16 (standard deviation, SD) in the bisglycinate group versus 0.80 ± 0.22 in the sulphate group; the difference was not statistically significant.

### 2.2. Statistics

The present study applied a statistical analysis based on the individual answers (yes/no) from the participating women instead of the percentual-based answers used earlier [10]. The MedCalc statistical software [12] was employed. Nonparametric statistics, the chi-square test (*χ*^2^ test) with Yate's correction, and Fisher's exact test were used for the comparison of GI complaints. The Bonferroni correction for multiple comparisons was applied. The significance level was set at *p* < 0.05. Data from the two studies are reported separately for each study and for both studies combined.

## 3. Results

### 3.1. GI Complaints at Inclusion in the Gentofte and Naestved Series

The frequency of GI complaints at inclusion is shown in Table 1. GI questionnaires were obtained from 404 women from Gentofte and 78 women from Naestved. In the Gentofte series, there was no significant difference in GI complaints between the women randomized into the four iron groups (results not shown), so the results of GI complaints in the four groups are combined in Table 1. In the Naestved series, the women randomized to ferrous bisglycinate had a slightly lower frequency of nausea than the women randomized to ferrous sulphate (Fisher's exact test, *p* = 0.047), but there were no significant differences between the frequencies of the other GI complaints, so the results of these two groups are also combined in Table 1. Women in the Gentofte series had a significantly lower mean body mass index (24 ± 3.2 kg/m^2^) than women in the Naestved series (26 ± 5.4 kg/m^2^) (Student's *t*-test for unpaired values *p* < 0.0001). Women in the Gentofte series had a slightly lower frequency of nausea and a significantly lower frequency of epigastric pain/pyrosis as well as total GI complaints compared to women in the Naestved series. At inclusion, there was no significant difference in the frequency of black stools: Gentofte series 34/404 vs. Naestved series 11/78 (Fisher's exact test *p* = 0.07).

In the two series combined, 21.2% of the answers (yes/no) to the total combined questions about GI discomfort were confirmative. The six most frequent complaints were meteorism, followed by flatulence, borborygmi, constipation, belching, and nausea (Table 1).

### 3.2. Gentofte Series

Table 2 shows the total combined frequencies of GI complaints in Gestational Weeks 32 + 39 and compares with the GI complaints at inclusion. In addition, there is a comparison between the frequencies of complaints in the four individual iron groups in Table 2.

Comparison between the frequency of GI complaints at inclusion and the GI complaints in Gestational Weeks 32 + 39 in the four iron groups (Table 2) showed that in all iron groups, there was a significant decline in the frequencies of nausea and vomiting, no change in epigastric pain/pyrosis, a significant increase in belching, a significant decrease in meteorism and borborygmi, no change in intestinal colic and flatulence, a significant increase in loose stools, and no change in constipation and use of laxatives.

A comparison of the frequencies of GI complaints in the four iron fumarate groups (Table 2) showed that the frequency of constipation was similar in the 20, 40, and 60 mg iron groups but significantly higher in the 80 mg iron group, and the use of laxatives increased steadily with the iron dose, being highest in the 80 mg iron group. The total combined frequency of complaints showed no consistency with the iron dose, being highest in the 20 mg iron group, lowest in the 60 mg iron group, and similar in the 40 and 80 mg iron fumarate groups (Table 2).

### 3.3. Naestved Series

Table 3 displays an overview of GI complaints during three time periods of pregnancy in the two iron groups. It appears that there was no significant difference between the ferrous bisglycinate group and the ferrous sulphate group at any of the time periods. However, the frequencies of total combined complaints were slightly but consistently higher in the ferrous sulphate group in all time periods, but the differences were not statistically significant, probably due to the low number of participants and observations (Table 3).

In Table 4, the frequencies of GI complaints during the three cumulated gestational observation periods (Weeks 27–28 + 36–37 + 39–40) have been combined and compared with the complaints at inclusion in each of the two iron groups. The ferrous bisglycinate group displayed a significant decrease in the frequency of intestinal colic as well as a decreased frequency of total combined complaints.

The ferrous sulphate group displayed a similar, significant decrease in nausea, borborygmi, and total combined complaints compared with the GI complaints at inclusion.

Comparison of GI complaints between the two iron groups showed no significant differences in the individual complaints but a significantly higher frequency of total combined complaints in the sulphate group compared to the bisglycinate group.

Comparison of ferrous bisglycinate, ferrous fumarate, and ferrous sulphate in doses being adequate to prevent ID in pregnant women.

In Table 5, we have compared the frequency of GI complaints during supplementation with different iron formulas (ferrous bisglycinate, ferrous fumarate, and ferrous sulphate) in doses of iron, which taken daily are sufficient to prevent ID and IDA in more than 95% of pregnant women [2, 9].

The frequency of epigastric pain/pyrosis was lowest in the 40 mg iron fumarate group and significantly higher in the 25 mg bisglycinate and the 50 mg sulphate group (Table 6). The frequency of belching and intestinal colic was highest in the fumarate group and significantly lower and similar in the bisglycinate and sulphate groups. The frequency of meteorism was lowest in the bisglycinate group and significantly higher and similar in the fumarate and sulphate groups. The total combined frequency of GI complaints was lowest in the bisglycinate group and significantly higher and similar in the fumarate and sulphate groups.

### 3.4. Frequency of Black Stools

At inclusion, the frequency of black stools was not significantly different in the Gentofte and Naestved studies, being 9.3% in the combined series (*n* = 482), most likely due to iron supplementation initiated in early pregnancy prior to inclusion in the studies.

In the Gentofte study, the 20 mg ferrous fumarate group displayed the same frequency of black stools as at inclusion. In the other iron groups, there was a significant increase in the frequency of black stools concomitant with the increase in iron dose (Table 6).

In the Naestved study, the frequency of black stools during gestation in the bisglycinate group was below the frequency at inclusion, although the difference was not significant (Table 7), while the sulphate group displayed a significant increase in the frequency of black stools compared to the frequency at inclusion. Consequently, the frequency of black stools during gestation was significantly lower in the bisglycinate group than in the sulphate group (Table 7).

The frequencies of black stools in women taking the three different iron formulas it does, which are adequate to prevent ID/IDA in pregnancy, are shown in Table 8. Clearly, the bisglycinate group had the lowest frequency of black stools, about 8%, while the fumarate and sulphate groups had similar frequencies, about 26%.

## 4. Discussion

In many countries, prophylactic oral iron supplementation is recommended during pregnancy to prevent ID and IDA. However, myths have been established among pregnant women concerning the possible GI side effects of iron [10], which may reduce compliance with iron supplementation. Low-dose iron supplements are usually assumed to have a low incidence of GI side effects, which are, however, often overestimated and exaggerated by psychological factors, and the fact that GI complaints in uncomplicated pregnancy per se are very frequent [6–8] makes it difficult to discriminate between pregnancy-induced physiological GI complaints and “side effect” complaints due to iron prophylaxis. These facts make it difficult to assess the true impact of iron supplementation on GI symptoms.

The present study reveals some of the difficulties associated with the evaluation of possible side effects of iron supplementation in pregnant women and, at the same time, yields results that can be used in the planning of iron prophylaxis in pregnancy care programmes. In the present-day setting, it will be considered unethical in Denmark to include a placebo-treated control group, so a “true” and statistically correct comparison between an iron and placebo group cannot be performed.

Uncomplicated single pregnancy is for various reasons frequently associated with an increased frequency of GI discomfort, as stated in the introduction [6–8]. It is therefore not surprising that the participants prior to inclusion in the study reported a high frequency of GI discomfort in early pregnancy (Table 1), being centred around intestinal gas problems and constipation.

The registration of GI complaints was based on a simple yes/no questionnaire reflecting the woman's subjective assessment of her symptoms, which could not be measured, quantified, or graduated on an objective scale. This might be improved by using a VAS scale to quantify symptoms.

The women were advised iron and vitamin supplements at the first antenatal visit according to the official pregnancy care recommendations by the Danish Health Authority [3], and the majority followed this advice [4, 5]. Thus, the registration of the baseline GI complaints may also be influenced and biased by the intake of iron supplements prior to inclusion, as reflected in the 9.3% frequency of black stools in the total series of women at the inclusion.

The Gentofte study [2] showed that in the groups taking 20–60 mg ferrous iron as fumarate, there was no significant association between the dose of ferrous fumarate and the frequency of GI side effects (Table 2). The only exception was that the highest iron dose of 80 mg was associated with a significantly higher frequency of constipation and use of laxatives. It should be taken into consideration that the frequency of constipation and the use of laxatives are interrelated. With the increasing use of laxatives, the true frequency of constipation may be underestimated, as we did not record how many of the women who answered “yes” to using laxatives also answered “yes” to having constipation.

There exists a distinct positive association between body mass index and the frequency of gastroesophageal reflux symptoms both in normal-weight and overweight women [13]. Women in the Gentofte series had a significantly lower frequency of epigastric pain/pyrosis than women in the Naestved series, which might partly be explained by the difference in mean body mass index between the two groups (see above).

Some of the results in the study were inconsistent; for example, the frequency of flatulence was similar in the 20, 40, and 80 mg fumarate groups but significantly lower in the 60 mg fumarate group (Table 2). This is probably a coincidental finding not being associated with this specific dose of iron.

From an overall point of view, there were only minor differences in GI complaints between the 20, 40, and 60 mg group, which appeared to have no clinically significant GI side effects, as none of the included women presented with GI complaints of such severity that it indicated either reduction of iron dose, change to an alternative iron formula, or discontinuation of iron supplement (Table 2). In contrast, the 80 mg fumarate group was associated with an increased frequency of constipation and use of laxatives. In this context, a dose of 80 mg of elemental iron cannot be considered a “low iron” dose, as it is within the range of the therapeutic iron doses of 100–200 mg iron/day used in the treatment of ID/IDA.

In the Naestved study [9], during supplementation with ferrous bisglycinate, there were significantly lower frequencies of intestinal colic and total complaints combined, compared to the frequencies at inclusion (Table 4), indicating that ferrous bisglycinate in the applied dose had no discernible GI side effects. The same results were found in women supplemented with ferrous sulphate, displaying a significantly lower frequency of nausea, borborygmi, and total complaints combined, indicating no clinically recognizable GI side effects. However, a comparison between the two iron formulas showed that the bisglycinate formula had a significantly lower frequency of total combined complaints, that is, most likely a better tolerance profile than the sulphate formula (Table 4). This is probably due to the lower dose of elemental iron of 25 mg combined with a higher bioavailability.

However, the changes in GI complaints from early to late pregnancy, that is, during iron supplementation, are also due to normal physiologic changes [6–8], which are independent of iron supplementation. In uncomplicated pregnancy, the frequency of nausea and vomiting is highest in early pregnancy and declines in late pregnancy, whereas the frequency of GI reflux is low in early pregnancy and usually increases in late pregnancy.

A comparison of the three different iron formulas in doses that are sufficient to prevent ID/IDA showed some differences in the frequencies of epigastric pain/pyrosis, belching, meteorism, and intestinal colic (Table 5). However, due to the lack of a placebo group, it is not possible to determine whether these differences may be caused by possible side effects of the iron formulas or are due to the normal physiological variations in GI complaints occurring during pregnancy. However, the frequency of total combined GI complaints was significantly lowest in the bisglycinate group and similar in the fumarate and sulphate groups, suggesting that ferrous bisglycinate is better tolerated than ferrous fumarate and ferrous sulphate.

Black stools are due to unabsorbed iron and indicate that either the iron formula is poorly absorbed due to low bioavailability and/or the iron formula is prescribed in a too large dose, which supersedes the intestinal iron absorption capacity. The frequency of black stools is closely associated with the iron dose (Table 6). Comparing the three different iron formulas, the ferrous bisglycinate group displayed the lowest frequency of black stools, probably due to the lower dose of elemental iron, while the frequency of black stools was higher and similar in the ferrous fumarate and ferrous sulphate groups.

## 5. Conclusions

This study shows that low-dose iron supplementation for pregnant women appears to have no clinically significant GI side effects. However, an iron dose above 60 mg of elemental ferrous iron as fumarate/day, for example, 80 mg/day, most likely causes an increased frequency of specific GI complaints. Comparing the three iron formulas in doses that can prevent ID and IDA, ferrous bisglycinate 25 mg iron had the most favourable GI side effect profile, while ferrous fumarate 40 mg iron and ferrous sulphate 50 mg iron had somewhat higher but similar GI side effect profiles. Ferrous bisglycinate may be considered for iron prophylaxis, especially in women experiencing GI side effects when taking conventional iron formulas.

## Figures and Tables

**Table 1:** Gastrointestinal complaints at inclusion in the oral iron supplementation protocols in the Gentofte and Naestved studies [2, 9].

**Gestation (weeks)**	**17–18 Gentofte inclusion**		**15–19 Naestved inclusion**		
	**n** ^a^	**% of women**	**n** ^a^	**% of women**	**Gentofte vs. Naestved ** ^**#**^ **p** ** value**
Complaints					
Nausea	68/404	16.8	23/78	29.5	0.012
Vomiting	30/404	7.4	10/78	12.8	ns
Epigastric pain/pyrosis	41/404	10.1	19/78	24.4	0.001
Belching	98/404	24.3	20/78	25.6	ns
Meteorism	160/404	39.6	39/78	50.0	ns
Borborygmi	135/404	16.3	30/78	38.5	ns
Intestinal colic	54/404	13.4	11/78	14.1	ns
Flatulence	154/404	38.1	35/78	44.9	ns
Loose stools	36/404	8.5	9/78	11.5	ns
Constipation	113/404	28.0	23/78	29.5	ns
Use of laxatives	16/404	4.0	2/78	2.6	ns
All complaints combined	905/4444	20.4	221/858	25.8	0.0005

Abbreviation: ns = nonsignificant.

^a^Figures show the number of women with complaints/number of women who answered the questionnaire.

^#^Fisher's exact test; significance level *p* < 0.05.

**Table 2:** Comparison between the combined total gastrointestinal complaints at inclusion and the combined total gastrointestinal complaints in Gestational Weeks 32 + 39 in each of the four iron supplementation groups (ferrous fumarate 20, 40, 60, and 80 mg of elemental iron/day) in the Gentofte study [2].

**Gestation (weeks)**	**17–18 inclusion**	32 + 39					**Inclusion vs. Weeks **32 + 39			
**Iron (mg/day)**		**20 mg**	**40 mg**	**60 mg**	**80 mg**	⁣^∗^**p value**	**20 mg**	**40 mg**	**60 mg**	**80 mg**
	**n** ^a^	**n** ^a^	**n** ^a^	**n** ^a^	**n** ^a^		^**#**^ **p** ** value**	^**#**^ **p** ** value**	^**#**^ **p** ** value**	^**#**^ **p** ** value**
Complaints										
Nausea	68/404	7/134	10/125	6/149	12/141	ns	0.0005	0.014	0.0001	0.018
	16.8%	5.2%	8.0%	4.0%	8.5%					
Vomiting	30/404	4/134	5/125	1/149	3/141	ns	ns	ns	0.001	0.023
	7.4%	3.0%	4.0%	0.7%	2.1%					
Epigastric pain/pyrosis	41/404	15/134	12/125	18/149	13/141	ns	ns	ns	ns	ns
	10.1%	11.2	9.6%	12.1%	9.2%					
Belching	98/404	70/134	63/125	69/149	83/141	ns	0.0001	0.0001	0.0001	0.0001
	24.3%	52.2%	50.4%	46.3%	58.9%					
Meteorism	160/404	35/134	33/125	38/149	35/141	ns	0.0005	0.008	0.003	0.002
	39.6%	26.1%	26.4%	25.5%	24.8%					
Borborygmi	135/404	22/134	21/125	21/149	26/141	ns	0.0001	0.0003	0.0001	0.0008
	33.4%	16.4%	16.8%	14.1%	18.4%					
Intestinal colic	54/404	20/134	19/125	19/149	24/141	ns	ns	ns	ns	ns
	13.4%	14.9%	15.%	12.8%	17.0%					
Flatulence	154/404	58/134	53/125	40/149	55/141	0.0001	ns	ns	0.0001	ns
	38.1%	43.3%	42.4%	26.8%	39.0%					
Loose stools	36/404	34/134	23/125	29/149	25/141	ns	0.0001	0.005	0.001	0.008
	8.9%	25.4%	18.4%	19.5%	17.7%					
Constipation	113/404	28/134	26/125	30/149	46/141	0.038	ns	ns	ns	ns
	28.0%	20.9%	20.8%	20.1%	32.6%					
Use of laxatives	16/404	0/134	5/125	7/149	10/141	0.026	0.016	ns	ns	ns
	4.0%	0%	4.0%	4.7%	7.1%					
All complaints combined	905/4444	393/1474	268/1375	268/1639	332/1551	0.003	0.0001	ns	0.0004	ns
	20.4%	26.7%	19.5%	16.4%	21.4%					

Abbreviation: ns = nonsignificant.

^a^Figures show the number of women with complaints/number of women who answered the questionnaire.

⁣^∗^*χ*^2^ test between the four iron groups in Weeks 32 + 39.

^#^Fisher's exact test between inclusion and Weeks 32 + 39 in the four iron groups; significance level *p* < 0.05.

**Table 3:** Gastrointestinal complaints at three time periods during gestation with iron supplementation in the two iron groups (ferrous bisglycinate 25 mg of elemental iron vs. ferrous sulphate 50 mg of elemental iron) in the Naestved study [9].

**Gestation (weeks)**	**27–28**			**36–37**			**39–40**		
**Iron (mg/day)**	**25 mg B** **n** ^a^	**50 mg S** **n** ^a^	^**#**^ **p** ** value**	**25 mg B** **n** ^a^	**50 mg S** **n** ^a^	^**#**^ **p** ** value**	**25 mg B** **n** ^a^	**50 mg S** **n** ^a^	^**#**^ **p** ** value**
Complaints									
Nausea	9/31	7/33	ns	4/28	6/31	ns	1/27	4/30	ns
Vomiting	2/31	4/33	ns	1/28	2/31	ns	1/27	1/30	ns
Epigastric pain/pyrosis	7/31	10/33	ns	8/28	13/31	ns	9/27	11/30	ns
Belching	6/31	9/33	ns	5/28	7/31	ns	7/27	9/30	ns
Meteorism	10/31	12/33	ns	6/28	10/31	ns	8/27	10/30	ns
Borborygmi	5/31	7/33	ns	4/28	4/31	ns	4/27	6/30	ns
Intestinal colic	3/31	4/33	ns	1/28	1/31	ns	0/26	1/30	ns
Flatulence	10/31	14/33	ns	7/28	10/31	ns	10/27	12/30	ns
Loose stools	2/31	3/33	ns	2/28	4/31	ns	3/27	6/30	ns
Constipation	6/31	12/33	ns	7/28	2/31	ns	7/27	6/30	ns
Use of laxatives	1/31	1/33	ns	0/28	0/31	ns	0/27	0/29	ns
All complaints combined	61/341 17.9%	83/363 22.9%	ns	43/280 15.4%	59/340 17.4%	ns	52/296 17.6%	68/329 20.47%	ns

*Note:* B = ferrous bisglycinate 25 mg of elemental iron, S = ferrous sulphate 50 mg of elemental iron.

Abbreviation: ns = nonsignificant.

^a^Figures show the number of women with complaints/number of women who answered the questionnaire.

^#^Fisher's exact test; significance level *p* < 0.05.

**Table 4:** Gastrointestinal complaints in the two iron supplementation groups (ferrous bisglycinate 25 mg of elemental iron vs. ferrous sulphate 50 mg of elemental iron) in the Naestved study [9]. The complaints at inclusion, prior to supplementation, are compared with the combined (Gestational Weeks 27–28 + 36–37 + 39–40) total complaints during supplementation.

**Gestation (weeks)**	**15–19 inclusion**	**27–40**		**15–19 inclusion**	**27–40**		**27–40**
**Iron (mg/day)**	**n** ^a^	**25 mg B** **n** ^a^	^**#**^ **p** ** value**	**n** ^a^	**50 mg S** **n** ^a^	^**#**^ **p** ** value**	**25 mg B vs. 50 mg S ** ^**#**^ **p** ** value**
Complaints							
Nausea	7/39	14/86	ns	16/39	17/94	0.008	ns
Vomiting	5/39	4/86	ns	5/39	7/94	ns	ns
Epigastric pain/pyrosis	8/39	24/86	ns	11/39	34/94	ns	ns
Belching	10/39	18/86	ns	10/39	25/94	ns	ns
Meteorism	18/39	24/86	ns	21/39	32/94	ns	ns
Borborygmi	12/39	13/86	ns	18/39	17/94	0.002	ns
Intestinal colic	7/39	4/86	0.035	4/39	6/94	ns	ns
Flatulence	18/39	27/86	ns	17/39	36/94	ns	ns
Loose stools	5/39	7/86	ns	4/39	13/94	ns	ns
Constipation	13/39	20/86	ns	10/39	20/94	ns	ns
Use of laxatives	0/39	1/86	ns	2/39	1/94	ns	ns
All complaints combined	103/429 24.0%	156/946 16.5%	0.001	118/429 27.5%	208/1034 20.1%	0.002	0.042

*Note:* B = ferrous bisglycinate, S = ferrous sulphate.

Abbreviation: ns = nonsignificant.

^a^Figures show the number of women with complaints/number of women who answered the questionnaire.

^#^Fisher's exact test; significance level *p* < 0.05.

**Table 5:** Comparison of gastrointestinal complaints during iron supplementation in pregnancy with three different iron formulas in doses being adequate to prevent iron deficiency: ferrous fumarate 40 mg of elemental iron from the Gentofte study [2], ferrous bisglycinate 25 mg of elemental iron, and ferrous sulphate 50 mg of elemental iron from the Naestved study [9].

**Gestation (weeks)**	32 + 39	**27–40**	**27–40**	⁣^∗^**p value**	^**#**^ **p** ** value**
**Iron (mg/day)**	**40 mg F** **n** ^a^	**25 mg B** **n** ^a^	**50 mg S** **n** ^a^		
Complaints					
Nausea	10/125	14/86	17/94	ns	
Vomiting	5/125	4/86	7/94	ns	
Epigastric pain/pyrosis	12/125	24/86^#^	34/94^#^	< 0.0001	ns^#^
	9.6%	27.9%	36.2%		
Belching	63/125	18/86^#^	25/94^#^	< 0.0001	ns^#^
	50.4%	20.9%	26.6%		
Meteorism	33/125^#^	10/86	32/94^#^	0.022	ns^#^
	26.4%	11.6%	34.0%		
Borborygmi	21/125	13/86	17/94	ns	
Intestinal colic	19/125	4/86^#^	6/94^#^	0.017	ns^#^
	15.2%	4.7%	6.4%		
Flatulence	53/125	27/86	36/94	ns	
Loose stools	23/125	7/86	13/94	ns	
Constipation	26/125	20/86	20/94	ns	
Use of laxatives	5/125	1/86	1/94	ns	
All complaints combined	268/1375	156/946^#^	208/1034^#^	ns	0.042^#^
	19.5%	16.5%	20.1%		

*Note:* B = ferrous bisglycinate 25 mg of elemental iron, F = ferrous fumarate 40 mg of elemental iron, S = ferrous sulphate 50 mg of elemental iron.

Abbreviation: ns = nonsignificant.

^a^Figures show the number of women with complaints/number of women who answered the questionnaire.

⁣^∗^*χ*^2^ test.

^#^Fisher's exact test; significance level *p* < 0.05.

**Table 6:** Comparison between the frequency of black stools at inclusion and in Gestational Weeks 32 + 39 in each of the four iron supplementation groups (ferrous fumarate 20, 40, 60, and 80 mg elemental iron/day) in the Gentofte study [2].

**Gestation (weeks)**	**17–18 inclusion**	32 + 39					**Inclusion vs. Weeks **32 + 39			
**Iron (mg/day)**		**20 mg**	**40 mg**	**60 mg**	**80 mg**	**Difference between groups**	**20 mg**	**40 mg**	**60 mg**	**80 mg**
Black stools *n*^a^	34/404 8.4%	10/134 7.5%	27/125 21.6%	87/149 58.4%	96/141 68.1%	⁣^∗^*p* value < 0.0001	^#^ *p* value ns	^#^ *p* value 0.0002	^#^ *p* value 0.00001	^#^ *p* value 0.00001

Abbreviation: ns = nonsignificant.

^a^Figures show the number of women with black stools/number of women who answered the questionnaire.

⁣^∗^*χ*^2^ test between the four iron groups in Weeks 32 + 39.

^#^Fisher's exact test; significance level *p* < 0.05.

**Table 7:** Comparison between the frequency of black stools at inclusion and in Gestational Weeks 27–40 in the two iron supplementation groups (ferrous bisglycinate 25 mg elemental iron and ferrous sulphate 50 mg elemental iron) in the Naestved study [9]. The results from the three gestational periods (Weeks 27–28 + 36–37 + 39–40) are combined.

**Gestation (weeks)**	**Inclusion 15–19**	**27–40**	**Inclusion vs. 27–40 B**	**27–40**	**Inclusion vs. 27–40 S**	**27–40 B vs. S**
**Iron (mg/day)**		**25 mg B**	**25 mg B**	**50 mg S**	**50 mg S**	**25 mg B vs. 50 mg S**
Black stools *n*^a^	11/78 14.1%	7/86 8.1%	^#^ *p* value ns	29/94 30.9%	^#^ *p* value 0.0112	^#^ *p* value 0.0002

*Note:* B = ferrous bisglycinate 25 mg of elemental iron, S = ferrous sulphate 50 mg of elemental iron.

Abbreviation: ns = nonsignificant.

^a^Figures show the number of women with black stools/number of women who answered the questionnaire.

^#^Fisher's exact test; significance level *p* < 0.05.

**Table 8:** Comparison between the frequency of black stools during pregnancy in women from the Gentofte and Naestved studies [2, 9] taking three different iron formulas in doses being adequate to prevent iron deficiency: ferrous fumarate 40 mg elemental iron, ferrous bisglycinate 25 mg elemental iron, and ferrous sulphate 50 mg elemental iron.

**Gestation (weeks)**	32 + 39	**27–40**	**27–40**		
**Iron (mg/day)**	**40 mg F**	**25 mg B**	**50 mg S**		
Black stools *n*^a^	27/125^#^ 21.6%	7/86 8.1%	29/94^#^ 30.9%	⁣^∗^*p* value 0.0008	^#^ *p* value ns

*Note:* B = ferrous bisglycinate 25 mg of elemental iron, F = ferrous fumarate 40 mg of elemental iron, S = ferrous sulphate 50 mg of elemental iron.

Abbreviation: ns = nonsignificant.

^a^Figures show the number of women with black stools/number of women who answered the questionnaire.

⁣^∗^*χ*^2^ test.

^#^Fisher's exact test; significance level *p* < 0.05.

## Data Availability

The data used to support the findings of this study references [6] are available from the corresponding author upon request.
